# Cross-Modal Complementarity Learning for Fish Feeding Intensity Recognition via Audio–Visual Fusion

**DOI:** 10.3390/ani15152245

**Published:** 2025-07-31

**Authors:** Jian Li, Yanan Wei, Wenkai Ma, Tan Wang

**Affiliations:** 1 School of Biology and Food Engineering, Fuyang Normal University, Fuyang 236037, China; 202201008@fynu.edu.cn; 2 Provincial Key Laboratory of Embryo Development and Reproductive Regulation, Fuyang 236037, China; 3 School of Information and Artificial Intelligence, Anhui Agricultural University, Hefei 230036, China; 4 Key Laboratory of Agricultural Sensors, Ministry of Agriculture and Rural Affairs, Hefei 230036, China

**Keywords:** fish feeding behavior, cross-modal fusion, attention mechanism, audio–visual, aquaculture monitoring

## Abstract

Accurate evaluation of fish feeding intensity is crucial for optimizing aquaculture efficiency and the healthy growth of fish. Previous methods mainly rely on single-modal approaches (e.g., audio or visual). However, the complex underwater environment makes single-modal monitoring methods face significant challenges: visual systems are severely affected by water turbidity, lighting conditions, and fish occlusion, while acoustic systems suffer from background noise. Although existing studies have attempted to combine acoustic and visual information, most adopt simple feature-level fusion strategies, which fail to fully explore the complementary advantages of the two modalities under different environmental conditions and lack dynamic evaluation mechanisms for modal reliability. To address these problems, we propose the Adaptive Cross-modal Attention Fusion Network (ACAF-Net), a cross-modal complementarity learning framework with a two-stage attention fusion mechanism: (1) a cross-modal enhancement stage that enriches individual representations through Low-rank Bilinear Pooling and learnable fusion weights; (2) an adaptive attention fusion stage that dynamically weights acoustic and visual features based on complementarity and environmental reliability. Our framework incorporates dimension alignment strategies and attention mechanisms to capture temporal–spatial complementarity between acoustic feeding signals and visual behavioral patterns. Extensive experiments demonstrate superior performance compared to single-modal and conventional fusion approaches, with 6.4% accuracy improvement. The results validate the effectiveness of exploiting cross-modal complementarity for underwater behavioral analysis and establish a foundation for intelligent aquaculture monitoring systems.

## Simple Summary

This study presents a novel cross-modal fusion method for accurate recognition of fish feeding intensity in complex underwater environments. Using acoustic and visual data from hydrophones and cameras, we develop a two-stage attention mechanism that adaptively combines complementary information from both modalities to overcome the limitations of single-modal approaches. The first stage enhances individual modal representations through cross-modal interactions, while the second stage dynamically adjusts fusion weights based on environmental conditions and modal reliability. Experimental results demonstrate that our method significantly outperforms existing single-modal and conventional fusion approaches, achieving superior accuracy in underwater scenarios. This work provides robust technical support for intelligent aquaculture monitoring and precision fish farming management.

## 1. Introduction

Fish play a crucial role in global food security and economic sustainability, with aquaculture representing one of the fastest-growing food production sectors worldwide [1,2]. Accurate recognition of fish feeding intensity is fundamental for optimizing aquaculture operations, as it directly influences feed conversion efficiency, growth rates, and overall fish health [3,4]. Traditional approaches to feeding intensity evaluation rely heavily on manual observation by experienced aquaculturists, who visually monitor fish behavior patterns and feeding responses [5]. However, this conventional method is labor-intensive, time-consuming, and prone to subjective interpretation, making it impractical for large-scale commercial operations. Moreover, continuous 24 h monitoring is often infeasible due to human limitations, leading to potential missed feeding opportunities and suboptimal feeding schedules that can significantly impact production efficiency and economic returns [6].

With the rapid advancement of machine learning and computer vision technologies, researchers have increasingly turned to automated visual monitoring systems for fish feeding behavior analysis. Vision-based approaches offer the advantage of providing rich spatial information about fish movement patterns, swimming trajectories, and feeding behaviors, enabling objective and quantitative recognition of feeding intensity [7]. For instance, Zhou et al. [8] developed a deep convolutional neural network to automatically detect fish feeding behaviors from underwater videos, achieving 90% accuracy in controlled environments. Similarly, Yang et al. [9] proposed a dual attention network based on the efficientNet-B2 model, which achieved the same results. Wang et al. [10] introduced a dynamic feeding method for aquaculture fish using a multi-task neural network with promising results in laboratory settings. However, visual monitoring systems face significant limitations in real-world aquaculture settings. Water turbidity, varying lighting conditions, and complex underwater environments severely compromise image quality and visibility [11]. Additionally, high fish density in commercial farming operations often leads to severe occlusion problems, making individual fish tracking and behavior recognition extremely challenging. These environmental constraints significantly limit the practical deployment of vision-only systems in commercial aquaculture operations.

As an alternative approach, acoustic monitoring using hydrophones has emerged as a promising solution for fish behavior analysis, offering several distinct advantages over visual methods. Acoustic systems are unaffected by visual impairments, such as water turbidity or lighting variations, can provide 360-degree omnidirectional monitoring, and are capable of detecting subtle feeding sounds, such as chewing and swallowing, which may not be visible in video recordings [12,13]. Recent studies have demonstrated the potential of acoustic monitoring in aquaculture applications. For example, Cui et al. [14] first transformed audio signals into log-mel-spectrograms and then fed them into a CNN-based model. The results demonstrate the potential of acoustic methods in aquaculture applications. Building upon this foundation, Du et al. [15] introduced GhostNet, a lightweight architecture specifically tailored for acoustic fish feeding behavior recognition that reduces computational requirements while maintaining high accuracy. Similarly, Iqbal et al. [13] proposed an innovative approach combining involutional neural networks with self-attention mechanisms for *Oplegnathus punctatus* feeding intensity classification, achieving state-of-the-art performance on log-mel-spectrograms. Despite these advantages, acoustic-only approaches also have limitations, including challenges in spatial localization and potential interference from background noise in complex aquatic environments [16]. This has motivated researchers to explore the complementary nature of acoustic and visual modalities.

Recognizing the complementary nature of acoustic and visual modalities, recent research has begun exploring multimodal fusion approaches for fish behavior analysis [4,17]. The fundamental premise is that acoustic and visual information capture different aspects of feeding behavior: visual data provides spatial context and movement patterns, while acoustic data offers temporal feeding signals and intensity information. Several pioneering studies have attempted to combine these modalities for enhanced fish monitoring. For example, Cui et al. [18] developed U-FFIA, a unified model capable of processing audio, visual, or combined audio–visual modalities, demonstrating improved accuracy compared to single-modal methods in controlled laboratory environments. Building upon this foundation, Du et al. [19] proposed the MFFFI model, which integrates three complementary data streams: audio (mel-spectrogram), video (RGB), and image sonar (IS) features to achieve more comprehensive feeding behavior analysis. Similarly, Gu et al. [20] introduced MMFINet, which enhances feeding intensity analysis accuracy and robustness by incorporating audio, video, and dissolved oxygen data, with experimental results confirming superior performance over single-modality approaches. Despite these promising developments, existing multimodal methods predominantly employ relatively simple fusion strategies, such as early concatenation of features or late score-level averaging, which fail to fully exploit the complex complementary relationships between modalities. These approaches treat acoustic and visual information as equally reliable across all environmental conditions, fundamentally ignoring the fact that modal quality and reliability vary significantly depending on dynamic factors, such as water conditions, lighting variations, and fish density fluctuations. Furthermore, current methods lack sophisticated adaptive mechanisms to dynamically adjust the contribution of each modality based on real-time environmental recognition, potentially leading to suboptimal fusion results when one modality becomes unreliable or compromised.

To address these limitations, we propose the Adaptive Cross-modal Attention Fusion Network (ACAF-Net), a novel cross-modal complementarity learning framework that intelligently fuses acoustic and visual information through a two-stage attention mechanism. Our approach first enhances individual modal representations by leveraging Low-rank Bilinear Pooling (LRBP) for cross-modal interactions, allowing each modality to benefit from complementary information provided by the other. Subsequently, an adaptive attention fusion stage dynamically weights the contribution of acoustic and visual features based on their reliability and complementarity under varying environmental conditions. This adaptive mechanism ensures optimal fusion performance across diverse underwater scenarios by automatically adjusting modal weights when environmental factors compromise the quality of specific modalities. The main contributions of this work are summarized as follows:

(1) We develop the first adaptive cross-modal attention fusion approach specifically designed for fish feeding intensity recognition, effectively addressing the limitations of existing single-modal and simple fusion methods.

(2) We introduce a sophisticated two-stage fusion architecture that first enhances modal representations through LRBP interactions and then adaptively weights modalities based on environmental reliability recognition and complementarity analysis.

(3) We conduct extensive experiments on a real-world fish feeding dataset, demonstrating significant performance improvements over state-of-the-art single-modal and conventional fusion approaches.

## 2. Materials and Methods

### 2.1. Dataset Acquisition and Materials

**Figure 1:**
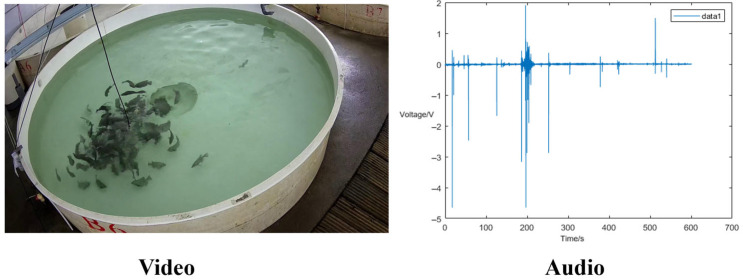
The example of the AV-FFIA dataset for fish feeding intensity analysis; video frames showing fish feeding behaviors; corresponding hydrophone signals with voltage variations indicating acoustic feeding activities.

In this study, we utilized the AV-FFIA dataset (as shown in Figure 1) from “Multimodal Fish Feeding Intensity Recognition in Aquaculture” [18], which provides synchronized acoustic and visual data specifically designed for fish feeding behavior analysis. The dataset features *Oplegnathus punctatus* as experimental subjects in a controlled recirculating aquaculture system. The experimental data were collected using a high-definition digital camera (1920 × 1080, 25 fps, Hikvision DS-2CD2T87E(D) WD-L, Hikvision, Hangzhou, China) and a high-frequency hydrophone (LST-DH01, Listen Technology, Changsha, China, sampling frequency: 256 kHz). Fish feeding sessions were conducted twice daily following standard aquaculture practices, with each recording session lasting 15 min, where feeding occurred from the second minute for approximately 3–10 min. This experimental design enabled clear temporal segmentation of feeding versus non-feeding periods. Feeding periods were defined as the time segments when pellets were actively provided (minutes 2–12), during which fish exhibited varying feeding responses. Non-feeding periods included both pre-feeding segments (0–2 min) and post-feeding segments (after minute 12) when no food was available, during which fish displayed natural swimming behaviors, resting, or other non-feeding activities. The feeding intensity was annotated by experienced aquaculture technicians into four categories based on standardized visual criteria [13,14] (as shown in Table 1), with corresponding acoustic data labeled according to the same temporal segments: “strong”, “medium”, “weak”, and “none”. The temporal validation protocol ensured that clips labeled as “strong”, “medium”, or “weak” were exclusively extracted from feeding periods, while the “none” category included both pre-feeding and post-feeding periods. Transition periods were carefully reviewed to avoid mislabeling boundary segments, ensuring precise separation between feeding and non-feeding behavioral states. Acoustic source validation was performed to ensure sounds were correctly attributed to feeding activities. We considered feeding-related sounds to include not only direct pellet consumption (cracking, jaw movements) but also associated feeding behaviors, such as water surface disturbance during food competition, increased swimming activity around feeding areas, and fish-to-fish interactions during feeding. These feeding-associated sounds showed strong temporal correlation (>0.85) with synchronized video recordings and exhibited characteristic frequency signatures in the 2–8 kHz range. Non-feeding sounds (background swimming, equipment noise) displayed distinct signatures: routine swimming sounds below 2 kHz with continuous patterns, and equipment noise with broadband steady-state characteristics. Expert validation on 1000 clips achieved 94.2% agreement on feeding-related sound attribution, with discrepant cases excluded from the dataset.

**Figure 2:**
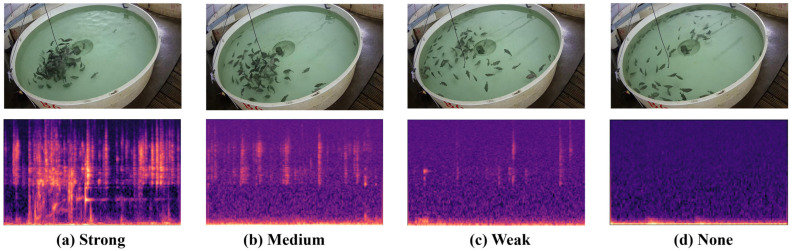
Representative examples of synchronized audio–visual data for fish feeding intensity recognition. Top row: video frames showing fish behavioral patterns for (**a**) strong, (**b**) medium, (**c**) weak, and (**d**) none feeding intensities. Bottom row: corresponding mel-spectrograms displaying acoustic signatures, where warmer colors (red/yellow) indicate higher energy and cooler colors (blue/purple) represent lower energy. Strong feeding shows high-energy patterns across 2–8 kHz frequency bands, while none periods exhibit minimal acoustic activity with primarily background noise.

The continuous recordings were segmented into 2 s clips, resulting in a total of 27,000 synchronized audio–visual pairs with balanced representation across all feeding intensity categories. The dataset was randomly partitioned into training (21,000 clips), validation (2800 clips), and testing (2800 clips) sets, maintaining consistent distribution across all feeding intensity categories to ensure robust evaluation of model performance. To ensure transparency and reproducibility, we note that the AV-FFIA dataset [18] is publicly available, enabling independent access and validation. We provide complete details of our preprocessing parameters (σ_s_ = 1.5, σ_r_ = 15), data partitioning strategy, and experimental configurations to ensure full reproducibility of our methodology and results. Figure 2 illustrates representative examples of synchronized audio–visual data across the four feeding intensity categories. The visual data clearly demonstrates the behavioral differences: strong feeding (a) shows multiple fish actively competing for food with high mobility and dense aggregation around feeding areas; medium feeding (b) displays fish moving toward food sources but maintaining some spacing; weak feeding (c) shows limited fish participation with sparse feeding activity; and none (d) exhibits fish in resting or swimming states without food-related behaviors. The corresponding mel-spectrograms reveal distinct acoustic signatures for each category. Strong feeding generates high-energy spectral patterns with a broad frequency distribution (2–8 kHz) due to intensive pellet cracking and multiple fish activities. Medium feeding shows moderate acoustic activity with a clear temporal structure. Weak feeding displays sporadic, low-amplitude spectral features, while the none periods contain primarily background noise from water circulation systems with minimal biological activity signatures. These examples demonstrate the complementary nature of audio–visual modalities, where visual data provides spatial behavioral context and acoustic data captures feeding intensity through sound characteristics that may not be visually apparent.

### 2.2. Audio and Visual Preprocessing

The original video frames with resolution 1920 × 1080 underwent a two-step preprocessing pipeline to achieve the required 224 × 224 input size. First, center cropping was applied to extract 1080 × 1080 square regions from the original frames, focusing on the central feeding area while maintaining the aspect ratio. Second, bilinear interpolation scaling was used to resize the cropped regions to 224 × 224 pixels. This approach preserves the spatial relationships and feeding behavior details while ensuring compatibility with standard deep learning architectures. The center cropping strategy was chosen to focus on the primary feeding zone, where fish activity is most concentrated during feeding sessions. To enhance model robustness and prevent overfitting, we applied comprehensive data augmentation strategies for both modalities. Video augmentation included the following: (1) horizontal flipping with 50% probability, (2) random rotation within ±15 degrees, (3) brightness adjustment with the factor range [0.8, 1.2], (4) contrast modification with the factor range [0.9, 1.1], and (5) random Gaussian blur with kernel size 3 × 3 and a probability of 30%. Audio augmentation applied to mel-spectrograms included the following: (1) time masking with a maximum 10% of time frames masked, (2) frequency masking with a maximum 8% of frequency bins masked, (3) random amplitude scaling with the factor range [0.9, 1.1], and (4) additive Gaussian noise with a signal-to-noise ratio > 20 dB. All augmentations were applied during training only, with validation and testing performed on original, unaugmented data to ensure fair evaluation. These augmentation strategies help improve the model’s generalization capability across different environmental conditions and viewing angles commonly encountered in aquaculture settings.

**Figure 3:**
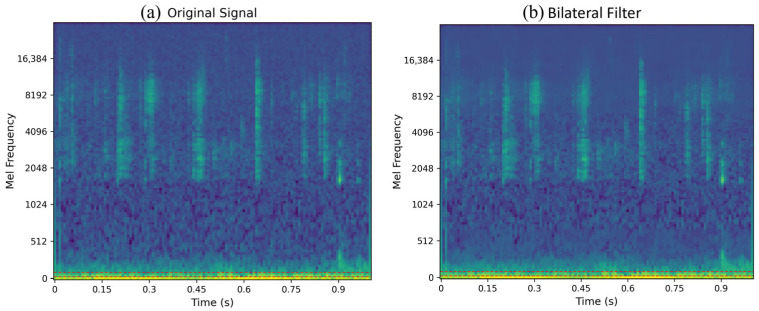
Mel-spectrogram denoising comparison: (**a**) original noisy mel-spectrogram and (**b**) denoised result using bilateral filtering. The original spectrogram exhibits extensive snowflake-pattern noise throughout the frequency domain, particularly in higher frequency bands. After filtering, the background noise is significantly reduced while fish feeding signals (vertical patterns) are well preserved, demonstrating the effectiveness of the proposed denoising approach.

For audio data processing, we first converted the raw acoustic signals into mel-spectrogram representations, which provide a time-frequency representation suitable for deep learning analysis. Due to the complex underwater environment, these mel-spectrograms contained significant background noise from water pumps and water flow systems that could interfere with feeding behavior detection. To address this challenge, we applied bilateral filtering [21] to the mel-spectrogram images as a denoising technique to remove environmental noise while preserving the essential acoustic characteristics of fish feeding behaviors (as shown in Figure 3).

The bilateral filter operates on the mel-spectrogram $S\in R^{M\times N}$, where M represents melfrequency bins and N represents time frames. The filtered output $\hat{S}\left(m,n\right)$ at each melfrequency m and time frame n is computed as Equation (1):

(1) $$\hat{S}\left(m,n\right)=\frac{1}{W\left(m,n\right)}\sum_{\left(i,j\right)\in \Omega }\;S\left(i,j\right)\cdot w_{s}\left(m,n,i,j\right)\cdot w_{r}\left(m,n,i,j\right)\;,$$

where Ω defines the spatial neighborhood around position $\left(m,n\right)$ and $W\left(m,n\right)$ is the normalization factor. The bilateral filter operates on two fundamental physical principles adapted for spectral domain processing. The spatial weight function $w_{s}\left(m,n,i,j\right)=exp\left(-\frac{(m-i{)}^{2}+(n-j{)}^{2}}{2\sigma_{s}^{2}}\right)$ represents geometric proximity in the mel-spectrogram domain, modeling the physical principle that neighboring spectral components share similar characteristics due to the continuous nature of acoustic phenomena. The range weight function $w_{r}\left(m,n,i,j\right)=exp\left(-\frac{|S(m,n)-S(i,j){|}^{2}}{2\sigma_{r}^{2}}\right)$ implements edge-preserving filtering based on spectral similarity, reflecting that genuine acoustic events create coherent spectral patterns with sharp boundaries, while noise exhibits random amplitude variations. The combined mechanism $w_{s}\cdot w_{r}$ ensures smoothing only occurs between spectrally similar neighbors within defined spatial proximity, effectively removing random noise while preserving coherent spectral structures characteristic of fish feeding events. The normalization factor ensures energy preservation: $W\left(m,n\right)=$ $\sum_{\left(i,j\right)\in \Omega }\;w_{s}\left(m,n,i,j\right)\cdot w_{r}\left(m,n,i,j\right)$, maintaining total spectral energy during filtering, which is crucial for subsequent feature extraction accuracy. This dual-weight mechanism enables the filter to smooth noise regions while preserving sharp spectral boundaries characteristic of fish feeding events. We empirically set $\sigma_{s}=1.5$ for spatial smoothing and $\sigma_{r}=15$ for range preservation based on the spectral characteristics of our underwater acoustic data.

We validated bilateral filtering effectiveness through SNR analysis, finding average improvements of 4.2 dB, 3.8 dB, and 2.1 dB for strong, medium, and weak feeding categories, respectively. We investigated alternative signal processing approaches: Adaptive Thresholding: magnitude-based thresholding (removing components below 10% of peak) improved strong/medium accuracy by 1.2% but degraded weak feeding recognition by 2.8% due to the removal of low-amplitude signals. SNR-adaptive Filtering: adjusting filter parameters based on local SNR (*σ_r_* = 20 for high-SNR, *σ_r_* = 10 for low-SNR regions) yielded minimal improvement (0.3%) with significantly increased computational complexity. Our empirical evaluation confirmed that fixed-parameter bilateral filtering provides an optimal balance between noise reduction and signal preservation across all feeding intensities, particularly for the challenging weak category, where maintaining low-amplitude signals is critical.

### 2.3. Methods

#### 2.3.1. Overall Architecture

**Figure 4:**
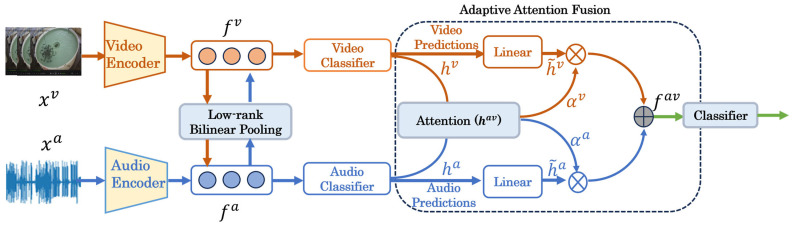
Detailed overview of the proposed ACAF-Net framework for fish feeding intensity recognition. The framework operates through three sequential stages: (1) feature extraction stage: individual modality encoders (MobileNetV2 for audio mel-spectrograms $x^{a}$, S3D for video frames $x^{v}$) extract domain-specific features $f^{a}\in R^{d}$ and $f^{v}\in R^{d}$, generating initial predictions $h^{a}$ and $h^{v}$ through respective classifiers. (2) Cross-modal enhancement stage: Low-rank Bilinear Pooling (LBP) facilitates bidirectional feature interactions through projection matrices $U^{a}$ and $U^{v}$ (rank r = 256), producing enhanced cross-modal features that are integrated with original features via learnable fusion weights $\alpha_{a}\;$*and* $\alpha_{v},$ resulting in enhanced audio and visual representations. (3) Adaptive attention fusion stage: enhanced features are processed through modality-specific MLPs to generate transformed predictions ${\tilde{h}}^{a}$ and ${\tilde{h}}^{v}$, which are concatenated and fed to an attention network that produces dynamic weights $\alpha^{a}$ and $\alpha^{v}$ based on environmental reliability assessment. The final prediction $f^{av}$ is computed through weighted fusion and classified via softmax to produce feeding intensity categories (none, weak, medium, strong).

Figure 4 illustrates the overall architecture of our proposed ACAF-Net framework for fish feeding intensity recognition. The method consists of three main components: (1) individual modality encoders for extracting audio and visual features, (2) Low-rank Bilinear Pooling for cross-modal enhancement, and (3) adaptive attention fusion for dynamic multimodal integration. Our ACAF-Net framework introduces several key technical innovations that distinguish it from conventional fusion approaches. First, the two-stage adaptive fusion architecture enables both cross-modal enhancement and dynamic weighting, where Stage 1 allows bidirectional feature interactions through Low-rank Bilinear Pooling before individual classification, and Stage 2 adaptively weights modality contributions based on environmental reliability. Second, the Low-rank Bilinear Pooling mechanism reduces computational complexity from *O(d_a_ × d_v_ × d_out_)* to *O((d_a_ + d_v_) × r)* while capturing complex non-linear cross-modal relationships through bidirectional enhancement with learnable fusion weights. Third, the environment-aware adaptive attention mechanism dynamically adjusts fusion weights based on modality reliability assessment, automatically emphasizing reliable modalities while de-emphasizing compromised ones through sigmoid-activated attention weights $\alpha^{a}$ with constraint $\alpha^{v}=1-\alpha^{a}$. Unlike conventional methods that employ static fusion strategies, our approach adapts to varying underwater conditions, where water turbidity may compromise visual quality or background noise may affect acoustic reliability. These technical innovations collectively enable superior performance (95.4% accuracy) with computational efficiency (11.8 M parameters) compared to existing specialized methods.

#### 2.3.2. Feature Extraction

The choice of feature extraction models is crucial for effective multimodal learning in fish feeding intensity recognition. In this study we choose pretrained MobileNetV2 as our audio encoder and pretrained S3D as our video encoder.

For audio feature extraction, we employ MobileNetV2 [22] to process the preprocessed mel-spectrogram images. MobileNetV2 was selected due to several key advantages that make it particularly suitable for underwater acoustic analysis: (1) computational efficiency: the depthwise separable convolutions significantly reduce computational complexity while maintaining feature extraction quality, which is essential for real-time aquaculture monitoring applications; (2) lightweight architecture: with only ~3.4 M parameters, MobileNetV2 enables deployment on resource-constrained edge devices commonly used in aquaculture environments; and (3) proven performance: as demonstrated in the original AV-FFIA dataset evaluation [18], MobileNetV2 achieved superior performance compared to other CNN architectures for audio-based feeding intensity classification, showing its effectiveness in capturing discriminative acoustic patterns from mel-spectrograms. The mel-spectrogram images are fed into MobileNetV2 to extract acoustic feature representations $f^{a}\in R^{d}$, which capture the temporal-frequency characteristics essential for distinguishing different feeding intensity levels through underwater acoustic signals.

For video feature extraction, we utilize S3D (separable 3D CNN) [23] to process the preprocessed video frame sequences. S3D was chosen as the optimal video encoder based on the following advantages: (1) spatio–temporal modeling: S3D effectively captures both spatial patterns and temporal dynamics in video sequences, which is crucial for understanding fish movement patterns and feeding behaviors that evolve over time; (2) separable 3D convolutions: the factorization of 3D convolutions into separate spatial (2D) and temporal (1D) components reduces computational cost while maintaining the ability to model complex spatio–temporal relationships; (3) superior performance: according to the AV-FFIA dataset benchmarking [18], S3D consistently outperformed other video analysis models including traditional 2D CNNs and standard 3D CNNs in fish feeding intensity classification tasks; and (4) efficient training: the separable convolution design allows for more stable training and better gradient flow compared to standard 3D convolutions, leading to improved convergence and performance. S3D processes video frame sequences to obtain visual feature representations $f^{v}\in R^{d}$, which encode spatial patterns and movement information related to fish feeding behaviors across temporal dimensions.

#### 2.3.3. Cross-Modal Enhancement via Low-Rank Bilinear Pooling

To exploit the complementary information between audio and visual modalities, we employ Low-rank Bilinear Pooling (LBP) for cross-modal feature enhancement. Traditional bilinear pooling suffers from high computational complexity and overfitting issues due to the large parameter space. Low-rank Bilinear Pooling addresses these limitations by constraining the interaction matrix to have a low rank, thereby reducing computational cost while maintaining the ability to capture meaningful cross-modal relationships.

Given the audio feature representation $f^{a}\in R^{d_{a}}$ and visual feature representation $f^{v}\in R^{d_{v}}$ extracted from MobileNetV2 and S3D, respectively, the Low-rank Bilinear Pooling operation can be formulated as Equation (2):

(2) $$LBP\left(fa,fv\right)={\left(U^{a}\right)}^{T}f^{a}⊙{\left(U^{v}\right)}^{T}f^{v},$$

where $U^{a}\in R^{d_{a}\times r}$ and $U^{v}\in R^{d_{v}\times r}$ are learned low-rank projection matrices, r is the rank parameter controlling the dimensionality of the interaction space, and ⊙ denotes element-wise multiplication. The traditional full-rank bilinear pooling would require a tensor $W\in R^{d_{a}\times d_{v}\times d_{out}}$ with $O\left(d_{a}\times d_{v}\times d_{out}\right)$ parameters, while our low-rank approach reduces this to $O\left(\left(d_{a}+d_{v}\right)\times r\right)$ parameters, where typically $r\ll \min\left(d_{a},d_{v}\right)$.

The cross-modal enhanced features are computed through bidirectional interactions as shown in Equations (3) and (4):

(3) $$f^{a\to v}=\mathrm{LBP}\left(f^{a},f^{v}\right)={\left(U^{a}\right)}^{T}f^{a}⊙{\left(U^{v}\right)}^{T}f^{v}$$

(4) $$f^{v\to a}=\mathrm{LBP}\left(f^{v},f^{a}\right)={\left(U^{v}\right)}^{T}f^{v}⊙{\left(U^{a}\right)}^{T}f^{a}$$

The enhanced features are obtained by incorporating the cross-modal information into the original features through residual connections with learnable fusion weights; the formula is shown in Equations (5) and (6):

(5) $$\tilde{f^{a}}=f^{a}+\alpha_{a}\cdot {\mathrm{MLP}}_{a}\left(f^{v\to a}\right)$$

(6) $$\tilde{f^{v}}=f^{v}+\alpha_{v}\cdot {\mathrm{MLP}}_{v}\left(f^{a\to v}\right),$$

where $\alpha_{a}$ and $\alpha_{v}$ are learnable scalar parameters that control the contribution of cross-modal information, and ${\mathrm{MLP}}_{a}$ and ${\mathrm{MLP}}_{v}$ are multi-layer perceptrons that transform the cross-modal features to match the dimensionality of the original features.

The low-rank constraint reduces the parameter count significantly, making the model more computationally efficient and less prone to overfitting. Despite the dimensionality reduction, LBP can still capture complex non-linear interactions between audio and visual features through the element-wise product operation. The symmetric design allows both modalities to benefit from each other’s information, enabling more comprehensive feature representations, while the residual connection design ensures stable gradient flow during backpropagation, facilitating better training dynamics. In our implementation, we set the rank parameter r = 256  to balance computational efficiency and representational capacity. The MLP layers consist of two fully connected layers with ReLU activation and dropout regularization. The fusion weights $\alpha_{a}$ and $\alpha_{v}$ are initialized to small positive values and learned during training to automatically determine the optimal contribution of cross-modal information. The enhanced features $\tilde{f^{a}}$ and $\tilde{f^{v}}$ are then fed to their respective classifiers to generate enhanced predictions $h^{a}$ and $h^{v}$, which serve as inputs to the subsequent adaptive attention fusion stage. This cross-modal enhancement ensures that each modality benefits from the complementary information provided by the other, leading to more discriminative feature representations for feeding intensity classification.

#### 2.3.4. Adaptive Attention Fusion

The second stage of our framework employs an adaptive attention mechanism to dynamically weight the contributions of audio and visual modalities based on their reliability and complementarity under varying environmental conditions. Unlike fixed fusion strategies that treat both modalities equally regardless of environmental factors, our adaptive approach automatically adjusts the modal weights to optimize fusion performance when specific modalities become unreliable due to factors such as water turbidity, lighting variations, or background noise interference.

Given the enhanced predictions $h^{a}\in R^{C}$ and $h^{v}\in R^{C}$ from the cross-modal enhancement stage, where C represents the number of feeding intensity classes, the adaptive attention mechanism first concatenates these predictions to form a joint representation, as shown in Equation (7):

(7) $$h^{av}=\left[h^{a};h^{v}\right]\in R^{2C}$$

The concatenated features are then processed through an attention network to generate modality-specific attention weights; the formula is shown in Equations (8) and (9):

(8) $$\alpha^{a}=\mathrm{AttentionNet}\left(h^{av}\right)\in R^{C}$$

(9) $$\alpha^{v}=1-\alpha^{a}\in R^{C},$$

where the AttentionNet consists of a multi-layer perceptron with the following architecture, shown in Equation (10):

(10) AttentionNet(x)=σ(Linear2(ReLU(Dropout(BatchNorm(Linear1(x)))))))

The network transforms the input from $R^{2C}$ to $R^{512}$ through the first linear layer, applies batch normalization for training stability, ReLU activation for non-linearity, and dropout with probability 0.5 for regularization. The second linear layer maps the features back to $R^{C}$, and the sigmoid function σ ensures that the attention weights are bounded between 0 and 1. The constraint $\alpha^{v}=1-\alpha^{a}$ ensures that the attention weights sum to one, maintaining a proper probability distribution over the modalities. This design allows the network to learn which modality is more reliable for each class prediction under current environmental conditions.

To address the dimensional mismatch between class-level attention weights ($R^{C}$) and feature-level representations, we transform the enhanced predictions through separate linear layers. The formula is shown in Equations (11) and (12):

(11) $$\tilde{h^{a}}=\sigma \left({\mathrm{Linear}}_{a}\left(h^{a}\right)\right)\in R^{D}$$

(12) $$\tilde{h^{v}}=\sigma \left({\mathrm{Linear}}_{v}\left(h^{v}\right)\right)\in R^{D},$$

where D = 512 is the transformed feature dimension, and ${\mathrm{Linear}}_{a}$ and ${\mathrm{Linear}}_{v}$ are modality-specific transformation layers that map from $R^{C}$ to $R^{D}$. The sigmoid activation ensures that the transformed features are normalized to the range [0, 1], facilitating stable fusion.

The final fused representation is computed through element-wise multiplication and addition in Equation (13):

(13) $$f^{av}=\alpha^{a}⊙\tilde{h^{a}}+\alpha^{v}⊙\tilde{h^{v}}\in R^{D},$$

where ⊙ denotes element-wise multiplication. This adaptive weighting allows the model to emphasize the more reliable modality while de-emphasizing the less reliable one based on the learned attention weights.

The fused features are then passed through a final classification layer to generate the feeding intensity predictions, as shown in Equation (14):

(14) $$p=\mathrm{Softmax}\left({\mathrm{Linear}}_{final}\left(f^{av}\right)\right)\in R^{C},$$

where ${\mathrm{Linear}}_{final}$ maps from $R^{D}$ to $R^{C}$, and the softmax function produces probability distributions over the four feeding intensity classes.

The adaptive attention mechanism provides several key advantages for underwater multimodal fusion. The learned attention weights automatically adapt to environmental conditions, giving higher weights to more reliable modalities when environmental factors compromise specific sensors. The global averaging strategy for dimension alignment preserves the relative importance relationships learned at the class level while enabling feature-level fusion. The end-to-end training allows the attention mechanism to learn optimal weighting strategies specific to fish feeding intensity recognition, rather than relying on hand-crafted fusion rules. Additionally, the attention weights provide interpretability by revealing which modality the model considers more reliable under different environmental conditions, offering valuable insights for aquaculture practitioners and system designers.

#### 2.3.5. Evaluation Metrics

To comprehensively evaluate the performance of our proposed ACAF-Net framework, we employ four widely used classification metrics: accuracy, precision, recall, and F1-score. These metrics are commonly adopted in classification tasks and have been extensively used in fish feeding intensity analysis studies, enabling fair comparison with existing methods in the literature.

*Accuracy* measures the overall correctness of the model by calculating the ratio of correctly classified samples to the total number of samples:

(15) $$Accuracy=\frac{\mathrm{TP}\;+\;\mathrm{TN}}{\mathrm{TP}+\mathrm{TN}\;+\mathrm{FP}+\;\mathrm{FN}}$$

where *TP*, *TN*, *FP*, and *FN* represent true positives, true negatives, false positives, and false negatives, respectively.

*Precision* quantifies the proportion of correctly identified positive predictions among all positive predictions made by the model:

(16) $$Precision=\frac{\mathrm{TP}}{\mathrm{TP}+\mathrm{FP}}$$

This metric is particularly important for feeding intensity recognition as it indicates how reliable the model’s positive feeding intensity predictions are.

*Recall* measures the proportion of actual positive cases that are correctly identified by the model:

(17) $$Recall=\frac{\mathrm{TP}}{\mathrm{TP}+\mathrm{FN}}$$

High recall is crucial for ensuring that feeding events are not missed, which is essential for accurate feeding behavior monitoring in aquaculture applications.

*F1-score* provides a harmonic mean of precision and recall, offering a balanced evaluation metric that considers both false positives and false negatives:

(18) $$F1\text{-}score=2\times \frac{\mathrm{Precision}\;x\;\mathrm{Recall}}{\mathrm{Precision}+\mathrm{Recall}}$$

The F1-score is particularly valuable when dealing with imbalanced datasets or when both precision and recall are equally important for the application.

These evaluation metrics enable comprehensive performance recognition and facilitate direct comparison with state-of-the-art methods in fish feeding intensity analysis. The use of multiple complementary metrics ensures robust evaluation across different aspects of model performance, providing insights into both overall accuracy and class-specific performance characteristics.

## 3. Results

### 3.1. Experimental Setup

We use an NVIDIA GeForce RTX 4090 chip with 24 GB of RAM (NVIDIA Corporation, Santa Clara, CA, USA) as the graphics card for core computation. The CPU model is Intel Core i9-14900XF, 5.80 GHz (Intel Corporation, Santa Clara, CA, USA). The version of the CUDA compiler is 11.8, the version of Python is 3.8.18, and the version of PyTorch used in the work is 2.1.1. All experiments were performed on this device. The model is trained using the Adam optimizer, with an initial learning rate of 1 × 10^−3^ and a learning rate schedule for 200 epochs with a batch size of 32. To prevent overfitting, we implement early stopping with a patience of 15 epochs and dropout regularization with a rate of 0.5. The MobileNetV2 and S3D backbone networks are initialized with pretrained weights from AudioSet [24] and Kinetics [25] datasets, respectively. Cross-entropy loss is used for the multi-class classification task. The rank parameter for Low-rank Bilinear Pooling is set to r = 256, and the attention network employs a single hidden layer with 512 units. All reported results are averaged over five independent runs with different random seeds to ensure statistical significance and reproducibility.

### 3.2. Overall Performance Comparison

We comprehensively evaluated the performance of our proposed ACAF-Net framework by comparing it against various single-modal approaches and multimodal fusion methods on the AV-FFIA dataset. The experimental results demonstrate the significant advantages of adaptive cross-modal fusion for fish feeding intensity recognition.

#### 3.2.1. Audio-Based Method Performance

Table 2 presents the performance comparison of different audio-based approaches for fish feeding intensity recognition. We evaluated several state-of-the-art audio classification models using mel-spectrogram representations processed through our bilateral filtering preprocessing pipeline. The results demonstrate the effectiveness of various architectures in capturing acoustic patterns from underwater feeding sounds.

MobileNetV2 achieved the highest performance among audio-only methods with 86.0% accuracy, demonstrating its effectiveness in capturing acoustic patterns from underwater feeding sounds. Despite MobileViT’s innovative hybrid architecture, which combines convolutional operations with self-attention mechanisms, it achieved 85.4% accuracy, slightly lower than MobileNetV2. This result highlights that MobileNetV2 provides the optimal balance between accuracy and computational efficiency for underwater acoustic analysis. Notably, our MobileNetV2 implementation achieved 86.0% accuracy, representing a significant improvement over the original implementation reported in the AV-FFIA dataset paper [18], which achieved 83.0% accuracy. This 3.0 percentage point improvement can be attributed to our enhanced preprocessing pipeline that incorporates bilateral filtering to effectively handle underwater noise contamination from water pumps and flow systems. The bilateral filter preserves feeding-related acoustic features while removing background interference, leading to more discriminative mel-spectrogram representations. The performance hierarchy among different architectures reveals interesting insights about their suitability for underwater acoustic analysis. MobileNetV2’s superior performance with only 2.2 M parameters (compared to MobileViT’s 5.7 M parameters) demonstrates that architectural efficiency is crucial for this specific task. The consistent performance across precision, recall, and F1-score metrics indicates that these audio-based methods achieve balanced classification performance across all four feeding intensity categories (strong, medium, weak, none).

#### 3.2.2. Video-Based Method Performance

Table 3 summarizes the performance of various video-based approaches for fish feeding intensity recognition. We compared both 2D CNN and 3D CNN architectures to evaluate their effectiveness in capturing spatio–temporal patterns of fish feeding behaviors. All methods were evaluated using preprocessed video frames that were cropped to 224 × 224 pixels and enhanced through data augmentation techniques.

S3D achieved the highest performance among video-only methods, with 89.0% accuracy, demonstrating superior capability in modeling spatio–temporal dynamics of fish feeding behaviors. The effectiveness of S3D can be attributed to its separable 3D convolutions that factorize spatio–temporal modeling into separate spatial (2D) and temporal (1D) components, enabling more efficient capture of feeding behavior dynamics while reducing computational complexity compared to standard 3D CNNs. The performance comparison reveals that modern 3D CNN architectures significantly outperform traditional approaches like C3D, which achieved only 84.6% accuracy despite having 78.4 M parameters. SlowFast achieved competitive performance with 88.3% accuracy but requires 34.6 M parameters, making it less suitable for practical deployment. I3D and TSM-ResNet50 also showed strong performance, but S3D provides the optimal balance between accuracy and computational efficiency with only 7.8 M parameters.

VideoMAE, despite being a recent vision transformer-based approach with 87.1 M parameters, achieved 86.5% accuracy, indicating that transformer architectures may not be as effective as specialized 3D CNNs for this specific underwater video analysis task. This suggests that the inductive biases inherent in convolutional architectures are particularly beneficial for modeling fish feeding behaviors.

#### 3.2.3. Audio–Visual-Based Method Performance

Table 4 presents the comprehensive comparison of multimodal fusion approaches for fish feeding intensity recognition. We evaluated representative fusion strategies from simple combination methods to sophisticated attention-based mechanisms, as well as state-of-the-art methods specifically designed for fish feeding analysis.

The results demonstrate a clear performance hierarchy among different fusion strategies. Basic fusion methods, including element-wise addition (90.5%), feature concatenation (91.2%), and score averaging (92.1%), provide baseline performance but fail to capture complex inter-modal relationships. Cross-attention achieved 93.4% accuracy by enabling cross-modal dependencies but uses static attention patterns, while specialized fish feeding methods, MMFINet (93.6%) and MFFFI (92.8%), showed competitive performance. Our proposed ACAF-Net achieved the highest accuracy of 95.4% with 11.8 M parameters, demonstrating superior performance while maintaining computational efficiency compared to methods like MMFINet (15.2 M parameters). Beyond accuracy improvements, ACAF-Net demonstrates excellent computational efficiency essential for real-world aquaculture applications. Our framework achieved a single clip inference time of 23.4 ms on NVIDIA GeForce RTX 4090 hardware, enabling real-time processing of 42.7 clips per second, which exceeds typical aquaculture monitoring requirements where feeding assessments occur every few minutes. The training process required 4.2 h for 200 epochs, while inference memory usage was only 2.1 GB, making deployment feasible on mid-range hardware commonly available in commercial aquaculture facilities. Preprocessing overhead remained minimal at 3.2 ms per video frame and 8.7 ms per 2 s audio clip, ensuring that the complete pipeline from raw sensor data to feeding intensity prediction can operate within real-time constraints. These performance characteristics, combined with superior accuracy, position ACAF-Net as a practical solution for intelligent feeding management systems in commercial aquaculture operations, where both accuracy and computational efficiency are critical for successful deployment.

**Figure 5:**
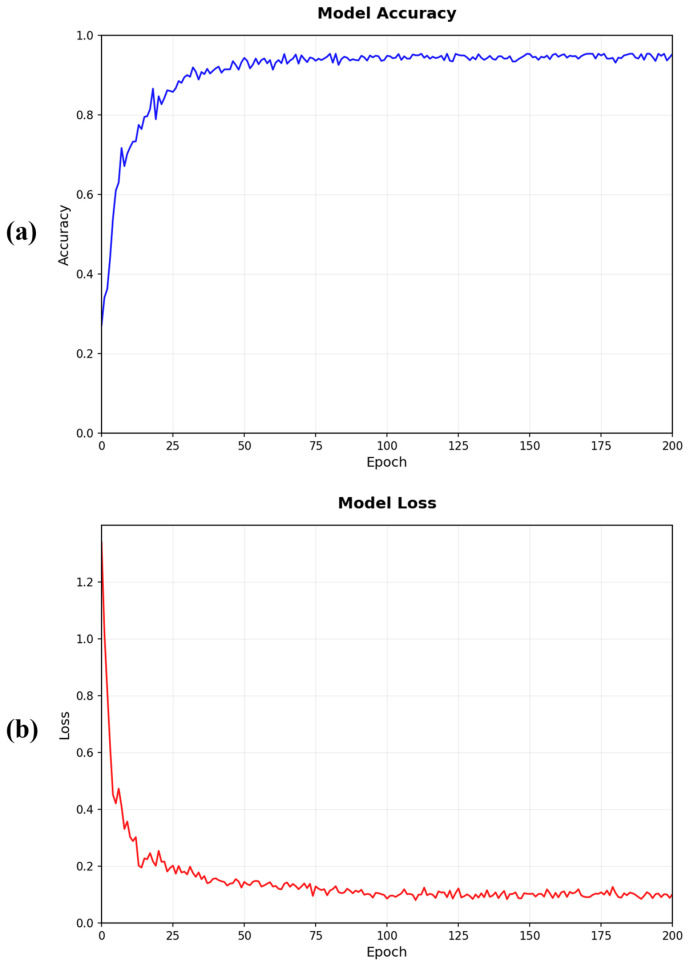
Training dynamics of ACAF-Net. (**a**) Training accuracy curve demonstrating rapid improvement from 30% to over 95% with stable convergence after epoch 100; (**b**) training loss curve showing smooth convergence from 1.25 to 0.08 over 200 epochs.

Figure 5 illustrates the training dynamics of ACAF-Net over 200 epochs. The training loss curve shows a smooth and consistent decrease from approximately 1.25 to 0.08, indicating stable optimization without significant oscillations. The training accuracy demonstrates a corresponding upward trend, rapidly increasing from 30% to over 85% within the first 25 epochs and then gradually converging to approximately 95.5% by epoch 100. The convergence behavior suggests that our two-stage fusion architecture facilitates effective gradient flow and stable learning dynamics. The smooth curves validate the robustness of our training strategy and the effectiveness of the learning rate schedule in achieving optimal model performance.

**Figure 6:**
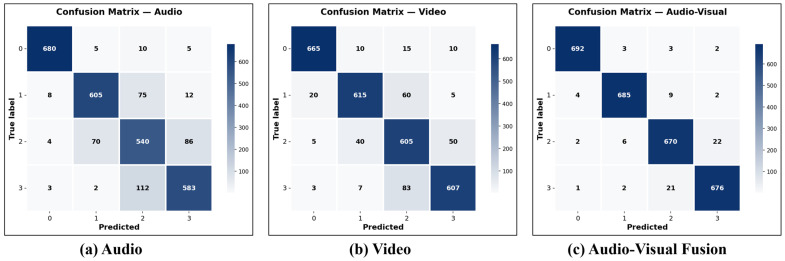
Confusion matrices for different modality approaches on fish feeding intensity classification. (**a**) Audio-only method using MobileNetV2; (**b**) video-only method using S3D; (**c**) audio–visual fusion using ACAF-Net. The matrices show classification results for four feeding intensity categories: 0 = none, 1 = strong, 2 = medium, and 3 = weak. ACAF-Net demonstrates superior performance with higher diagonal values and reduced misclassifications between adjacent intensity levels.

Figure 6 presents the confusion matrices for audio-only, video-only, and audio–visual fusion methods, providing detailed insights into classification performance across different feeding intensity categories (0 = none, 1 = strong, 2 = medium, 3 = weak). The audio-only method shows reasonable performance with diagonal values of 680, 605, 540, and 583 for classes 0–3, respectively, but exhibits notable confusion between adjacent intensity levels, particularly between medium and weak categories (112 misclassifications). The video-only method demonstrates improved performance with diagonal values of 665, 615, 605, and 607, showing better discrimination between classes but still experiencing some confusion in the medium category (83 misclassifications to weak). Our ACAF-Net fusion approach achieves the highest diagonal values of 692, 685, 670, and 676, with significantly reduced off-diagonal misclassifications. The fusion method shows particularly improved performance in distinguishing between the none and strong categories (only 4 misclassifications compared to 8 and 20 for audio and video, respectively) and between medium and weak categories (21 misclassifications compared to 112 and 83). This analysis demonstrates that the adaptive fusion strategy effectively combines the strengths of both modalities while mitigating individual modal limitations, resulting in more accurate and balanced classification across all feeding intensity levels.

#### 3.2.4. Ablation Study

To validate the effectiveness of each component in our proposed ACAF-Net framework, we conducted comprehensive ablation studies by systematically removing or replacing key modules. Table 5 presents the detailed ablation results using a clear component-wise analysis format, demonstrating the individual contribution of each component to the overall performance.

The ablation results demonstrate the individual and combined contributions of each component. The baseline method using simple feature concatenation achieves 91.2% accuracy. Adding the bilateral filter (BF) alone provides a 1.1% improvement to 92.3%, validating the effectiveness of our enhanced preprocessing pipeline in handling underwater noise contamination. Low-rank Bilinear Pooling (LBP) independently contributes a 1.9% improvement (93.1%), highlighting the importance of cross-modal feature interactions. The combination of LBP and BF achieves 93.8% accuracy, demonstrating synergistic effects between preprocessing enhancement and cross-modal learning.

Adaptive attention fusion (AAF) alone improves performance to 94.1%, showing the value of dynamic modality weighting. When combined with BF, the performance reaches 94.6%, while the combination with LBP achieves 94.9%. The full ACAF-Net model, incorporating all components (LBP + BF + AAF), achieves the highest accuracy of 95.4%, representing a 4.2% improvement over the baseline. This systematic analysis validates that each component contributes meaningfully to the overall performance, with positive interactions between different modules. The results confirm the necessity of our two-stage architecture design, where preprocessing enhancement (BF), cross-modal feature learning (LBP), and adaptive fusion (AAF) work together to achieve optimal performance for fish feeding intensity recognition in complex underwater environments.

## 4. Discussion

### 4.1. Key Findings and Technical Contributions

Our experimental results demonstrate that ACAF-Net achieves significant improvements in fish feeding intensity recognition, with 95.4% accuracy representing a 6.4% improvement over the best single-modal method and a 1.8% improvement over existing multimodal approaches. The substantial performance gap between single-modal methods (86.0% audio, 89.0% video) and our fusion approach empirically validates the complementary nature of acoustic and visual modalities in underwater scenarios. The confusion matrix analysis reveals that fusion particularly improves classification accuracy for adjacent feeding intensity levels, addressing a key challenge in practical aquaculture monitoring where subtle intensity differences are crucial for optimal feeding management.

The bilateral filtering approach for mel-spectrogram denoising proved crucial for underwater acoustic analysis, providing consistent improvements across all tested architectures and contributing to a 3.0% improvement in MobileNetV2 performance compared to the original study. This finding suggests that domain-specific preprocessing strategies are essential for practical deployment in complex aquaculture environments where water pumps and flow systems create significant background interference. The 0.8% improvement of adaptive attention over fixed attention mechanisms demonstrates the practical value of underwater monitoring systems, particularly relevant for real-world deployment where water conditions, lighting, and fish behavior patterns vary dynamically.

ACAF-Net achieves superior performance with computational efficiency, requiring fewer parameters (11.8 M) compared to specialized methods like MMFINet (93.6%, 15.2 M parameters). This efficiency advantage stems from our Low-rank Bilinear Pooling approach, which captures meaningful cross-modal interactions while maintaining computational tractability. The consistent performance improvements across different backbone architectures suggest that our fusion strategy is architecture-agnostic and could potentially benefit other multimodal applications beyond fish feeding recognition. Our two-stage fusion architecture contributes to the broader field of multimodal learning by demonstrating the importance of both cross-modal enhancement and adaptive fusion.

### 4.2. Limitations and Future Perspectives

Several limitations should be acknowledged in our current work. Our evaluation is limited to a single fish species (*Oplegnathus punctatus*) in controlled laboratory conditions, and future work should validate the approach across different fish species, tank configurations, and environmental conditions to establish broader generalizability. The current approach requires synchronized audio–visual data collection, which may increase system complexity compared to single-modal solutions. While our method demonstrates computational efficiency in parameter count, the two-stage architecture introduces additional computational steps that may require optimization for strict real-time requirements in commercial operations.

The ACAF-Net framework addresses critical practical requirements for real-world aquaculture implementation. Our system demonstrates excellent scalability potential through its lightweight architecture (11.8 M parameters) and efficient processing capabilities (23.4 ms inference time per clip), enabling deployment across facilities ranging from single research tanks to large commercial operations with hundreds of monitoring points. The framework supports flexible deployment strategies, including distributed edge computing, where individual tank clusters operate autonomous monitoring units that communicate feeding assessments to centralized management systems for coordinated feeding decisions. The modest hardware requirements (2.1 GB inference memory) ensure cost-effective widespread installation, while real-time processing capabilities enable immediate feeding adjustments critical for optimizing feed conversion ratios in commercial settings. The standardized preprocessing pipeline minimizes site-specific calibration requirements, reducing deployment complexity across diverse aquaculture environments. These characteristics position ACAF-Net as a practical foundation for industrial-scale intelligent feeding management systems. The proposed method addresses a critical need in precision aquaculture by enabling automated, continuous monitoring of feeding behaviors, which could significantly reduce feed waste, improve fish welfare, and optimize production efficiency. The interpretable attention weights provide valuable insights for aquaculture practitioners to understand feeding patterns and environmental influences. Beyond aquaculture, the principles of adaptive multimodal fusion demonstrated in this work could extend to other environmental monitoring applications where sensor reliability varies with environmental conditions, such as wildlife monitoring, marine ecosystem recognition, or agricultural automation.

Future research directions include developing incremental learning capabilities to extend the framework to new fish species while preserving knowledge of previously learned species, enabling practical deployment across diverse aquaculture operations without retraining from scratch [38]. Additional directions include extending the approach to handle missing modalities or temporal misalignment between sensors through semi-supervised learning approaches, developing model compression techniques for enhanced real-time performance, and exploring continuous intensity estimation rather than discrete categorization. The incremental learning approach would be particularly valuable for commercial deployment, allowing aquaculture facilities to gradually expand monitoring capabilities to different species while maintaining existing system performance, thereby reducing implementation costs and deployment complexity in multi-species operations. The relatively low computational requirements make the approach feasible for edge computing devices, enabling real-time local processing without requiring cloud connectivity, while the adaptive nature reduces the need for frequent manual calibration as the system automatically adjusts to changing environmental conditions.

## 5. Conclusions

In this paper, we introduced ACAF-Net, a novel adaptive cross-modal attention fusion framework for fish feeding intensity recognition, capable of dynamically weighting acoustic and visual modalities based on environmental conditions. The proposed framework effectively addresses the challenges of underwater multimodal analysis through a two-stage architecture that combines cross-modal enhancement via Low-rank Bilinear Pooling with adaptive attention fusion. Extensive experiments on the AV-FFIA dataset demonstrate the superior performance of ACAF-Net compared to state-of-the-art methods, achieving 95.4% accuracy while maintaining computational efficiency. The key contributions of this work include the enhanced preprocessing pipeline with bilateral filtering for underwater noise handling, the Low-rank Bilinear Pooling mechanism for efficient cross-modal interactions, and the adaptive attention mechanism that automatically adjusts to varying environmental conditions. The framework achieves practical deployment potential with reduced computational requirements compared to existing specialized methods. In future work, we plan to extend the validation across different fish species and diverse environmental conditions to establish broader generalizability. We will focus on developing semi-supervised approaches to handle missing modalities or temporal misalignment between sensors, which is common in real-world aquaculture deployments. Additionally, we aim to optimize the framework for enhanced real-time performance through model compression techniques and explore continuous intensity estimation rather than discrete categorization. Finally, we plan to conduct long-term field studies in commercial aquaculture facilities to validate the robustness and practical utility of the proposed approach under varying seasonal and operational conditions.

## Figures and Tables

**Table 1:** Descriptions of different fish feeding intensities (FFI).

FFI	Description
None	Fish do not respond to food
Weak	Fish eat only pellets that fall directly
	in front of them but do not move to take food
Medium	Fish move to take the food, but return to
	their original positions
Strong	Fish move freely between food items and
	consume all the available food

**Table 2:** Performance comparison of audio-based methods.

Audio Method	Accuracy (%)	Precision (%)	Recall (%)	F1-Score (%)	Parameters (M)
ResNet18 [26]	82.3	81.8	82.1	81.9	11.2
ResNet50 [27]	84.1	83.7	83.9	83.8	23.5
EfficientNet-B0 [28]	83.6	83.2	83.4	83.3	4.0
MobileViT [29]	85.4	85.1	85.3	85.2	5.7
VGG16 [30]	81.7	81.2	81.5	81.3	134.3
MobileNetV1 [31]	84.8	84.3	84.6	84.4	3.2
**MobileNetV2**	**86.0**	**85.8**	**85.9**	**85.7**	**2.2**

**Table 3:** Performance comparison of video-based methods.

Video Method	Accuracy (%)	Precision (%)	Recall (%)	F1-Score (%)	Parameters (M)
ResNet3D-18 [32]	85.4	85.1	85.3	85.2	33.2
I3D [33]	87.2	86.8	87.0	86.9	12.3
C3D [34]	84.6	84.2	84.4	84.3	78.4
TSM-ResNet50 [35]	87.8	87.4	87.6	87.5	24.3
SlowFast [36]	88.3	87.9	88.1	88.0	34.6
VideoMAE [37]	86.5	86.1	86.3	86.2	87.1
**S3D**	**89.0**	**88.7**	**89.2**	**88.8**	**7.8**

**Table 4:** Performance comparison of multimodal fusion methods.

Fusion Method	Accuracy (%)	Precision (%)	Recall (%)	F1-Score (%)	Parameters (M)
Element-Wise Addition	90.5	90.1	90.3	90.2	11.0
Feature Concatenation	91.2	90.8	91.0	90.9	11.2
Score Averaging	92.1	91.6	91.8	91.7	11.0
Cross-Attention	93.4	92.9	93.2	93.0	12.8
MMFINet [20]	93.6	93.1	93.4	93.2	15.2
MFFFI [19]	92.8	92.3	92.6	92.4	13.5
**ACAF-Net (Ours)**	**95.4**	**95.1**	**95.3**	**95.2**	**11.8**

**Table 5:** Ablation study results.

Models	LBP	BF	AAF	Acc.
Baseline	*X*	*X*	*X*	91.2
+BF	*X*	✓	*X*	92.3
+LBP	✓	*X*	*X*	93.1
+LBP+BF	✓	✓	*X*	93.8
+AAF	*X*	*X*	✓	94.1
+BF+AAF	*X*	✓	✓	94.6
+LBP+AAF	✓	*X*	✓	94.9
**ACAF-Net (Full)**	✓	✓	✓	95.4

LBP: Low-rank Bilinear Pooling; BF: bilateral filter; AAF: adaptive attention fusion; *X* indicates the component is not used and ✓ indicates the component is used.

## Data Availability

The AV-FFIA dataset used in this study is publicly available from Cui et al. [18] (https://github.com/FishMaster93/U-FFIA, accessed on 28 July 2025). The dataset is provided under CC BY. Our preprocessing code, model implementations, and experimental configurations are available from the corresponding authors upon reasonable request.
